# Supplementary material for: Dop1 enhances conspecific olfactory attraction by inhibiting miR-9a maturation in locusts
Source: Nat Commun. 2018 Mar 22;9:1193. doi: 10.1038/s41467-018-03437-z (PMC5864846; doi:10.1038/s41467-018-03437-z)
Supplement: Supplementary file 1 — Supplementary Information(PDF 1517 kb) [file 41467_2018_3437_MOESM1_ESM.pdf]

Supplementary Information

Dop1 enhances conspecific olfactory preference by inhibiting miR-9a maturation in locusts

Guo et al.

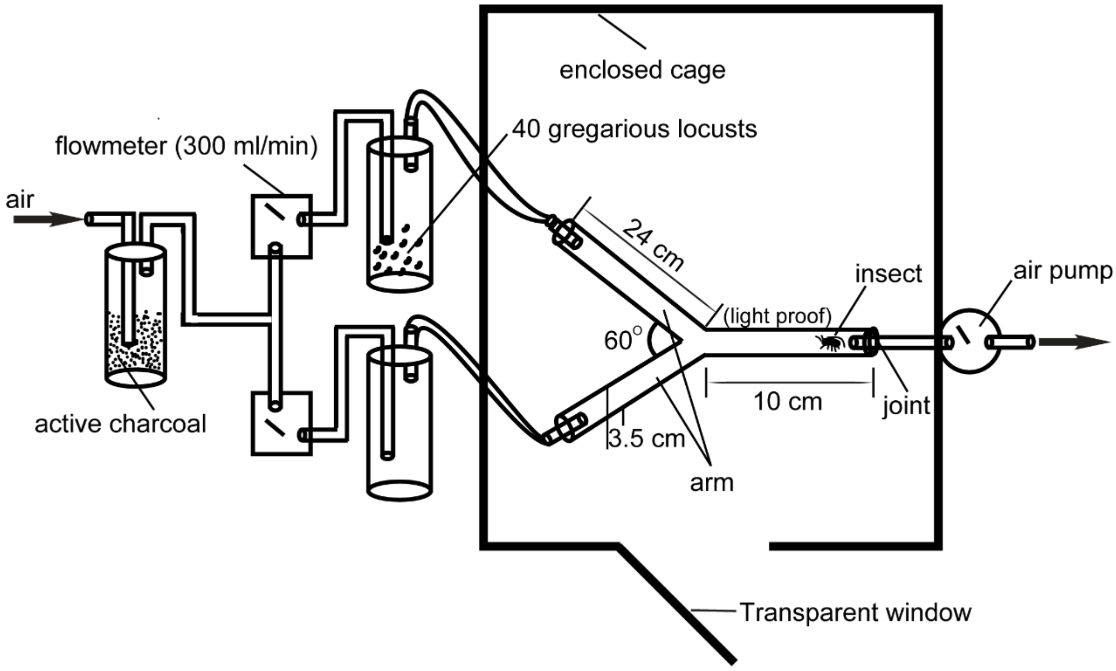

Supplementary Figure 1. The diagram of the Y-maze as used for testing olfactory preferences of the locusts in the present study.

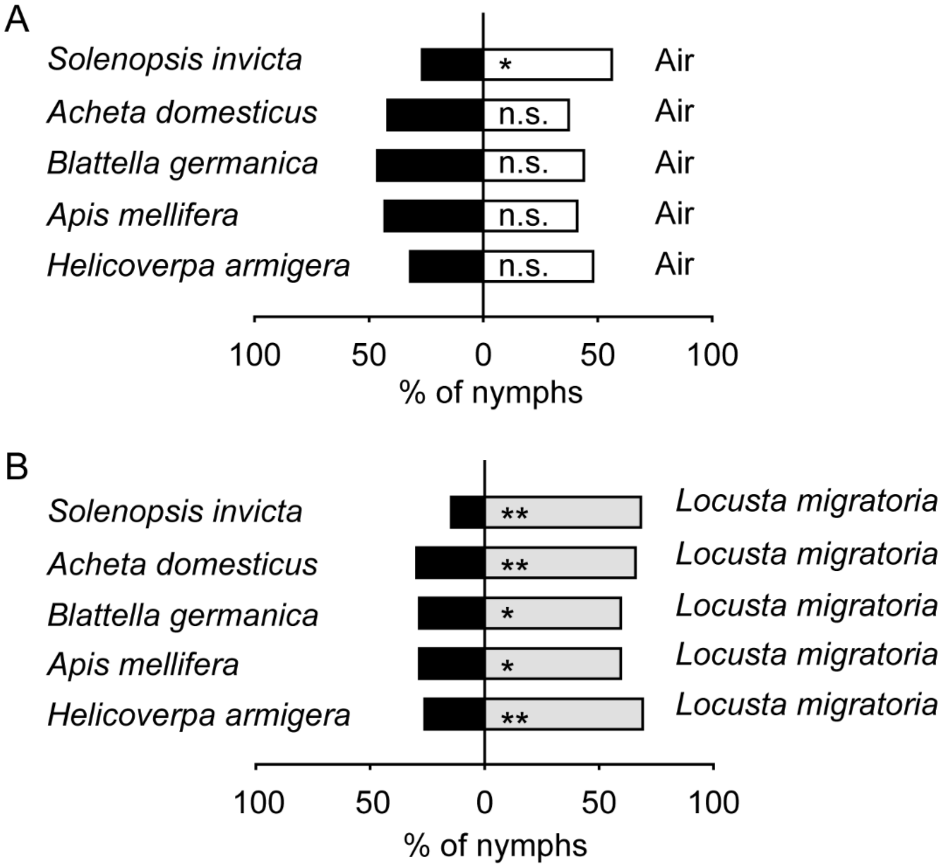

Supplementary Figure 2. The gregarious locusts are specifically attracted to the conspecific gregarious volatiles. (A) The olfactory responses of gregarious locusts to air and the volatiles of *Solenopsis invicta* (n = 41), *Acheta domesticus* (n = 49), *Blattella germanica* (n = 41), *Apis mellifera* (n = 44), *Helicoverpa armigera* (n = 50). (B) The olfactory responses of gregarious locusts to gregarious volatiles and the volatiles of *Solenopsis invicta* (n = 41), *Acheta domesticus* (n = 47), *Blattella germanica* (n = 41), *Apis mellifera* (n = 42), *Helicoverpa armigera* (n = 42). The asterisks (* and **) inside the strip indicate the significant differences of comparing individual numbers in each arm after *G*-test for goodness-of-fit (*, $P < 0.05$; **, $P < 0.01$; n.s., not significant).

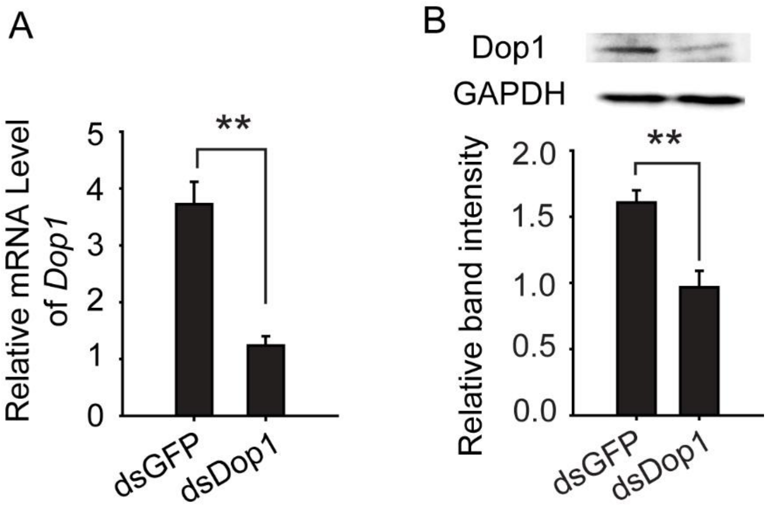

Supplementary Figure 3. The effects of Dop1 RNAi knockdown in the locust brain. (A) The mRNA level of *Dop1* after injection of dsDop1 in the locust brain. (B) The protein level of Dop1 after injection of dsDop1 in the locust brain. Injection of dsDop1 induced knockdown of Dop1 was used to validate the antibody specificity, and dsGFP injection was used as the control. The mRNA and protein level of Dop1 are analyzed by Student's *t*-test and presented as the mean ± SEM (mRNA, n = 8; protein, n = 6). **, $P < 0.01$.

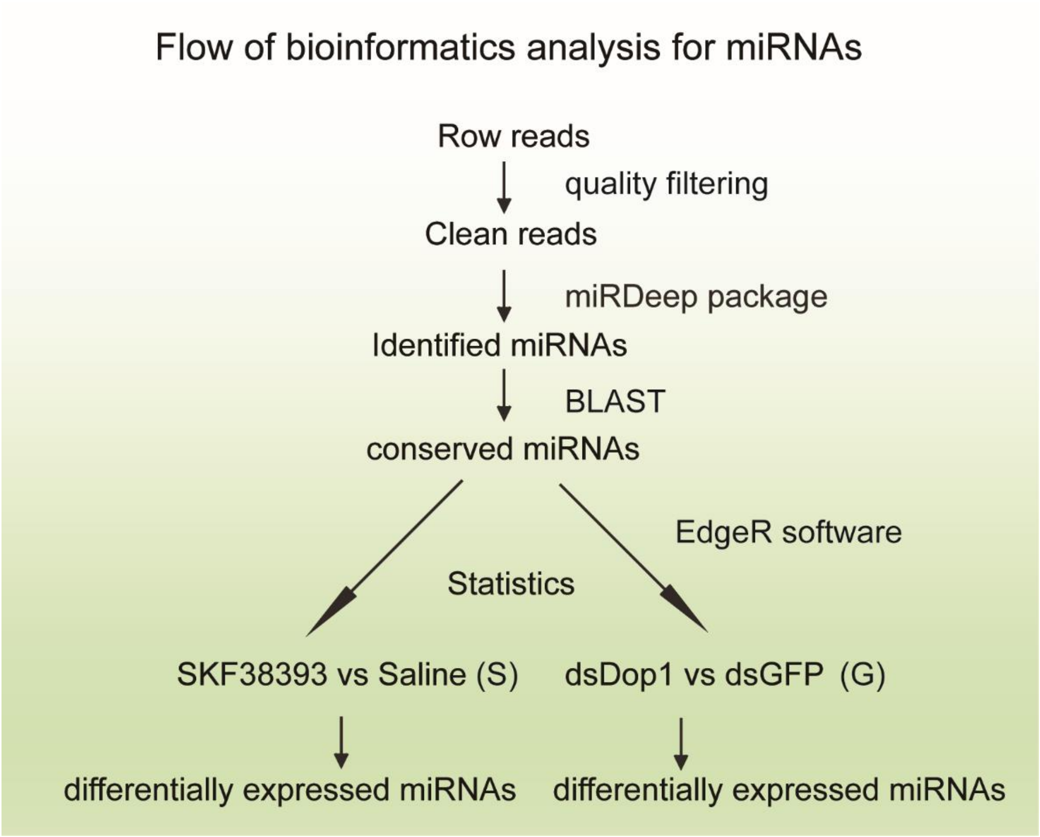

Supplementary Figure 4. The flow of analysis for differentially expressed miRNAs. The small RNA transcriptomes were determined by high-throughput sequencing, and the locust miRNAs were identified using *L.migratoria* genome and the miRDeep package. Second, all known arthropod miRNAs deposited in the latest release of miRBase were used to identify the locust miRNA homologs by homology searches. The differentially expressed miRNAs were calculated by EdgeR software. Abbreviations: S, solitarious; G, gregarious.

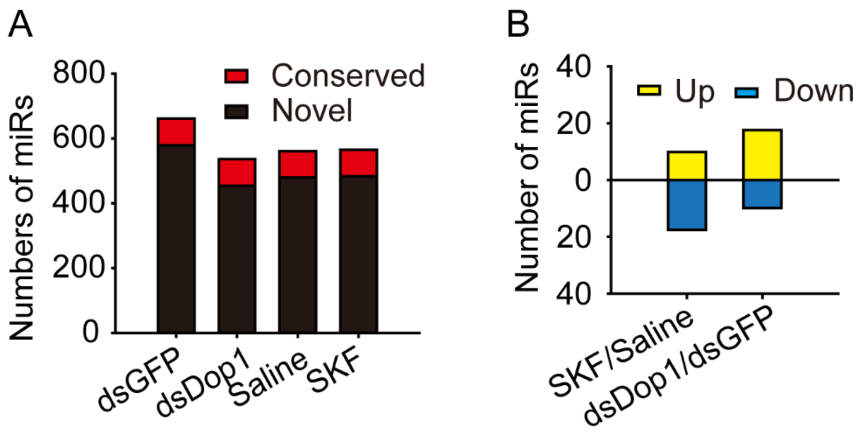

Supplementary Figure 5. The total miRNAs and differentially expressed miRNAs in different groups. (A) The numbers of conserved and novel miRNAs identified in the brains with Dop1 activation and inhibition, respectively. The red bars indicate the number of conserved miRNAs, and the black bars indicate the number of novel miRNAs. (B) The numbers of conservative miRNAs expressed differentially after activation and inhibition of Dop1. Abbreviations: SKF, SKF38393.

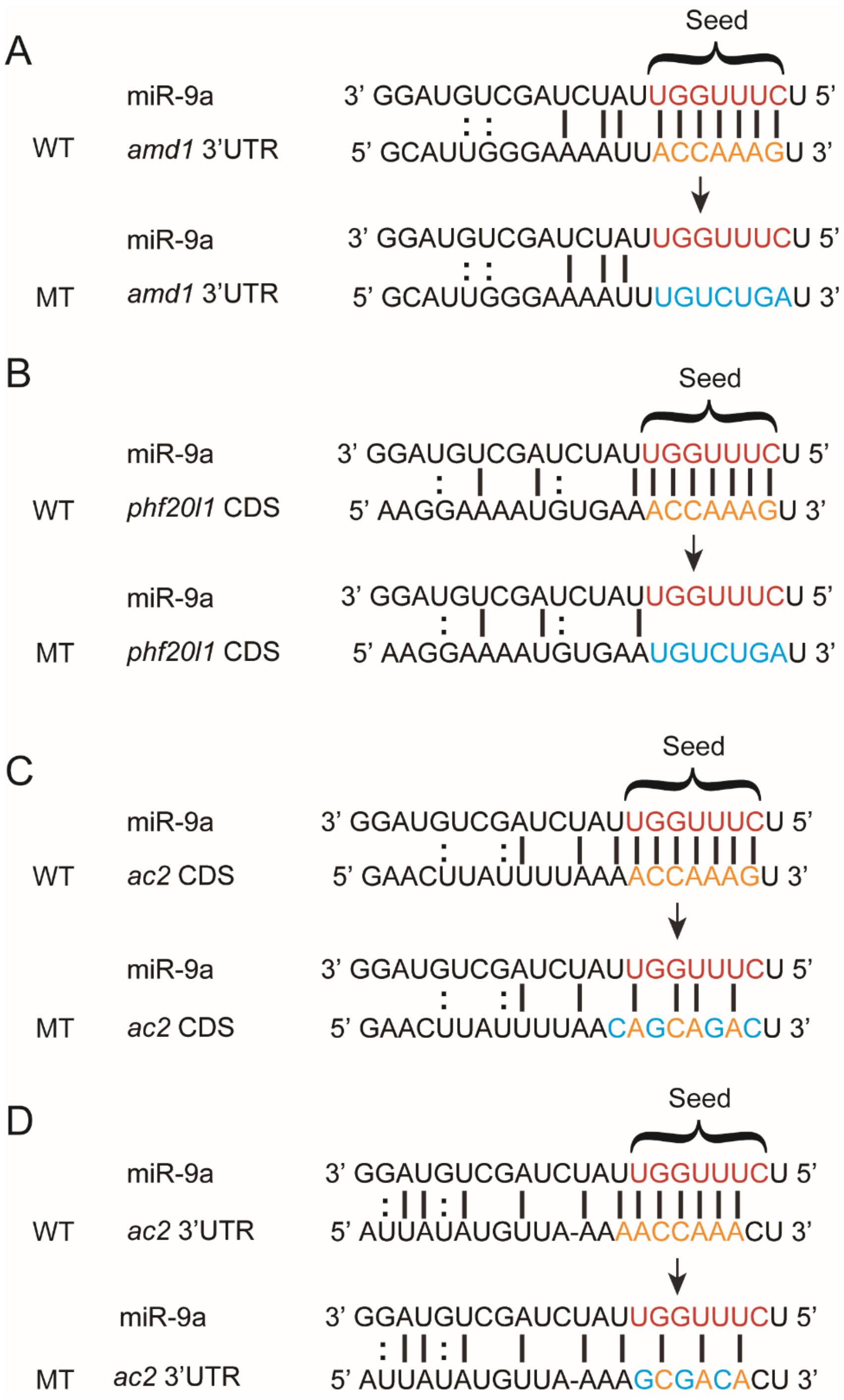

Supplementary Figure 6. The mutation sites in the putative target genes. (A) The mutation sites in 3’UTR region of *amd1* gene. (B) The mutation sites in CDS region of *phf20l1* gene. (C) The mutation sites in CDS region of *ac2* gene. (D) The mutation sites in 3’-UTR region of *ac2* gene. The point mutations in the putative target sites (WT, orange) were engineered in the region complementary to the miR-9a seed sequence (red). Those point mutations of MT sequences are indicated in blue.

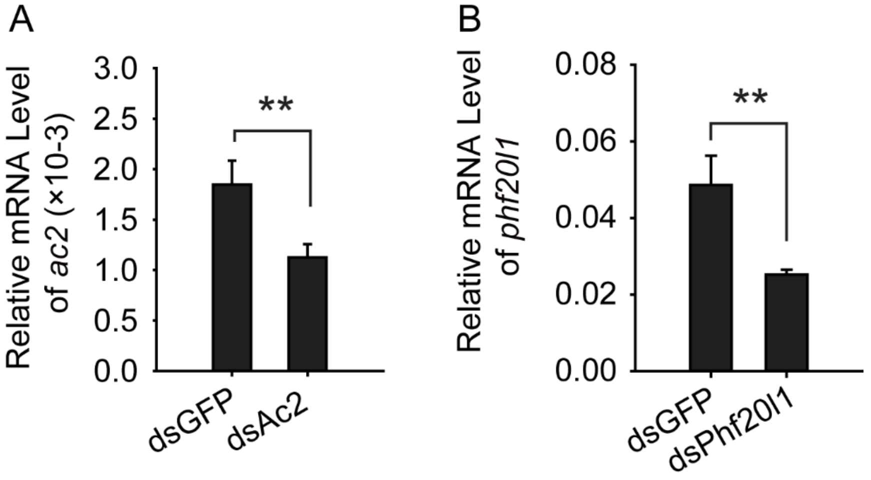

Supplementary Figure 7. The effects of gene RNAi knockdown in the locust brain. (A) The mRNA level of *ac2* after injection of dsAc2 in the brain of gregarious locusts. (B) The mRNA level of *phf20l1* after injection of dsPhf20l1 in the brain of solitarious locusts. The mRNA level of *phf20l1* and *ac2* are analyzed by Student'*s* *t*-test and presented as the mean ± SEM (n = 8). **, $P < 0.01$.

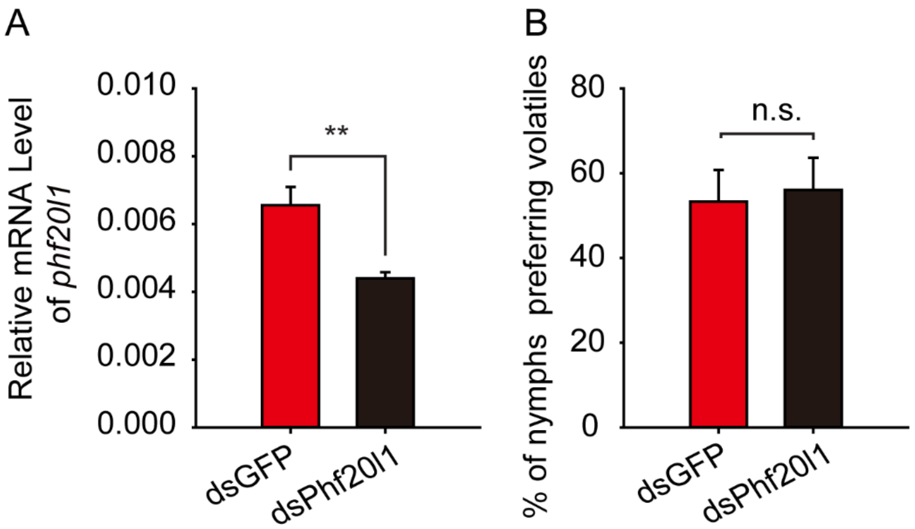

Supplementary Figure 8. Knockdown of *phf20l1* did not change the olfactory preference of the gregarious locusts. (A) The mRNA level of *phf20l1* after injection of dsPhf20l1 in the brain of the gregarious locusts. The mRNA level of phf20l1 are presented as the mean ± SEM (n = 8). (B) The olfactory responses of the gregarious locusts after *phf20l1* RNAi knockdown (n = 45 and 41). The data are shown as proportion (*p*) ± SE. The asterisks outside the strip indicate the significant difference between controls and the treatments through Student's *t*-test and *G*-test for independence. n.s., not significant; **, $P < 0.01$.

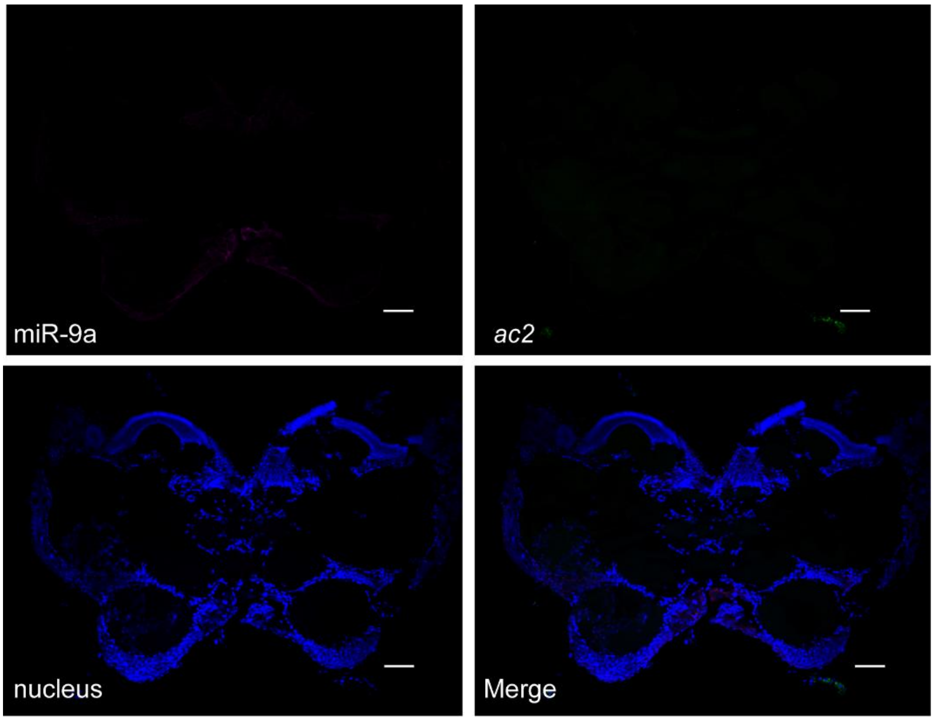

Supplementary Figure 9. The negative control for co-localization of miR-9a and *ac2* in the locust brain. The nucleus of locust brain is labeled by Hoechst33342 (Life Technology). The images were visualized using an LSM 710 confocal fluorescence microscope (Zeiss) at magnifications of 10×. Scale bar represents 100 μm.

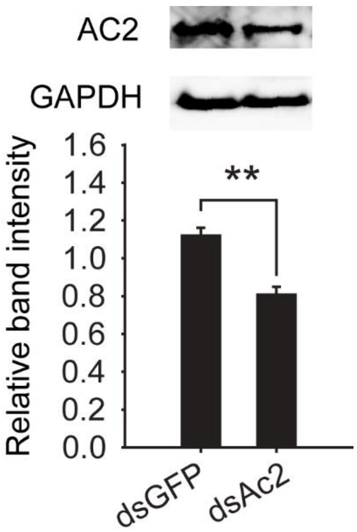

Supplementary Figure 10. The effects of AC2 RNAi knockdown in the locust brain. The protein level of AC2 after injection of dsAc2 in the locust brain. Injection of dsAc2 induced knockdown of AC2 was used to validate the antibody specificity, and dsGFP injection was used a control. The protein level of AC2 are analyzed by Student'*s* *t*-test and presented as the mean ± SEM (n = 6). **, $P < 0.01$.

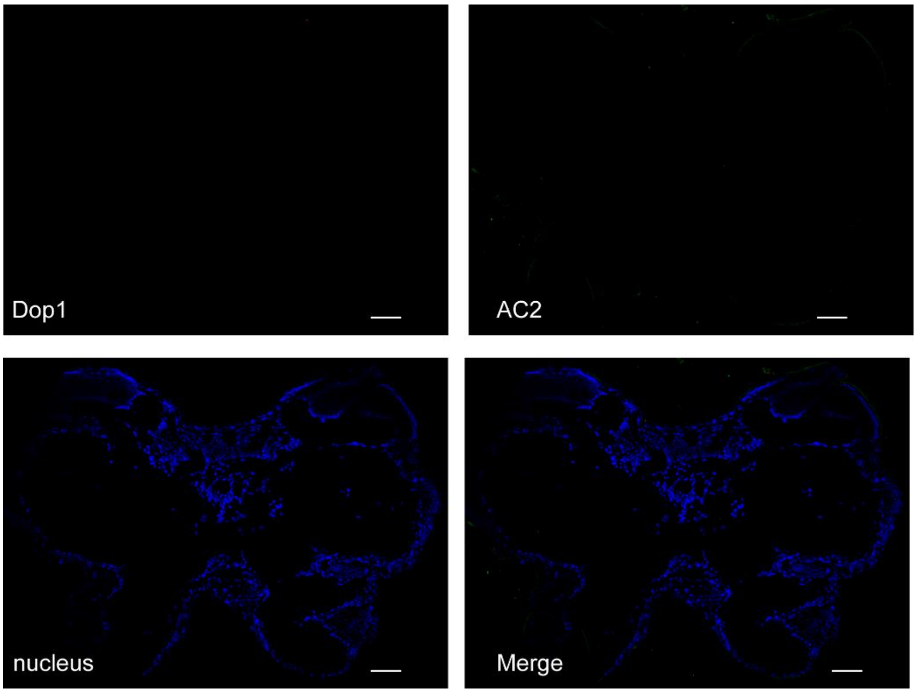

Supplementary Figure 11. The negative control for co-localization of Dop1 and AC2 in the locust brain. The nucleus of locust brain is labeled by Hoechst33342 (Life Technology). The images were visualized using an LSM 710 confocal fluorescence microscope (Zeiss) at magnifications of 10×. Scale bar represents 100 μm.

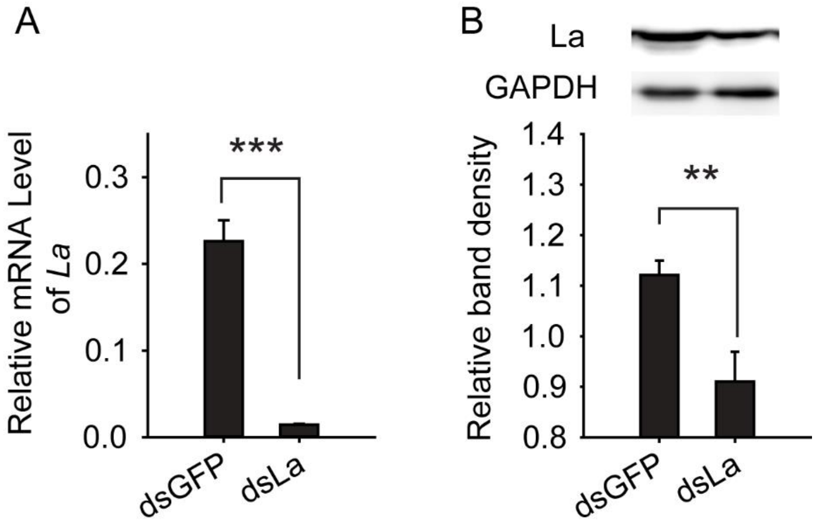

Supplementary Figure 12. The effects of La protein RNAi knockdown in the locust brain. (A) The mRNA level of *La* after injection of dsLa in the locust brain. (B) The protein level of La protein after injection of dsLa in the locust brain. Injection of dsLa induced knockdown of La protein was used to validate the antibody specificity, and dsGFP injection was used a control. The mRNA and protein level of La protein are analyzed by Student'*s t*-test and presented as the mean ± SEM (mRNA, n = 8; protein, n = 6). **, $P < 0.01$; ***, $P < 0.001$.

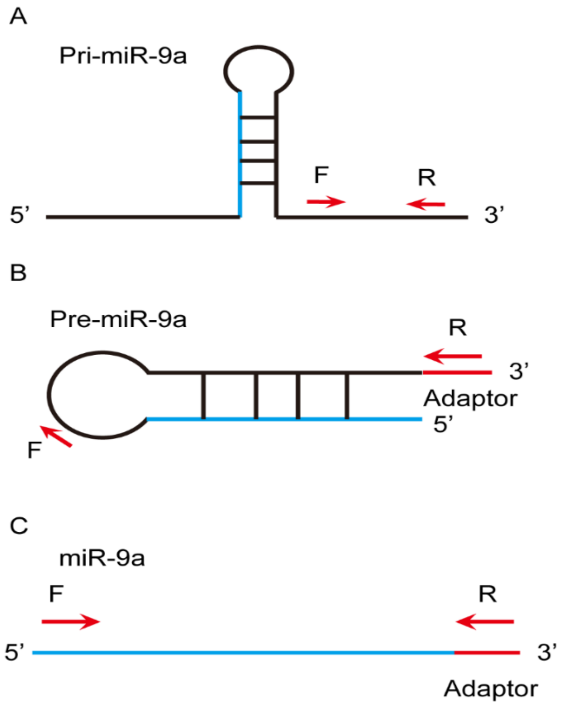

Supplementary Figure 13. Primer design for selective quantification of pri-miR-9a, pre-miR-9a, and miR-9a. (A) The specific forward (F) and reverse (R) primers of pri-miR-9a were designed in the part of pri-miR-9a not including pre-miR-9a. (B) The specific forward primer of pre-miR-9a was designed in the loop region, and the reverse primer complementary to the adaptor was provided in the qPCR kit. (C) The specific forward primer of miR-9a was designed according to the sequence of miR-9a, including the seed region, and the reverse primer complementary to the adaptor was provided in the qPCR kit.

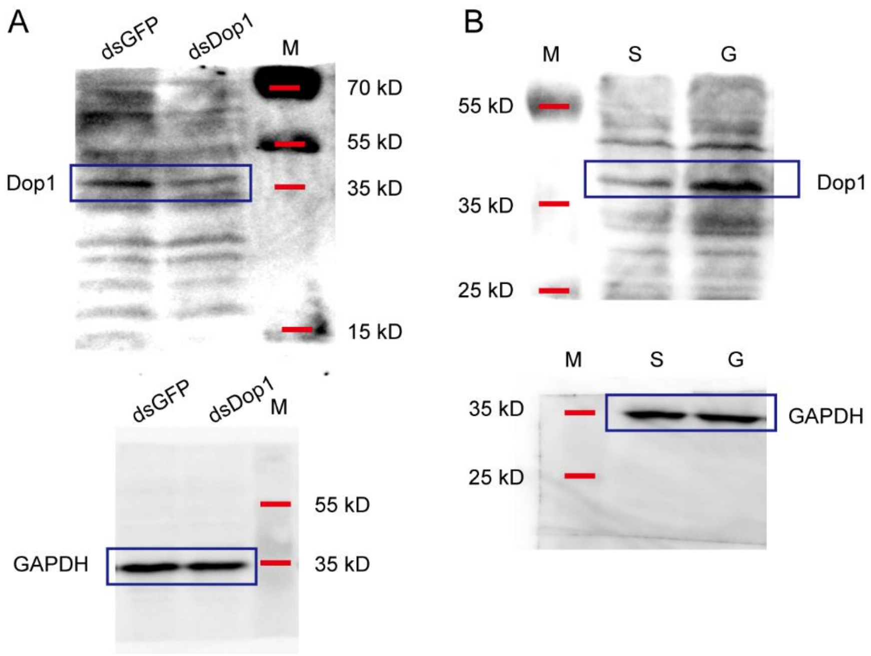

Supplementary Figure 14. The full-size western blot scans of Dop1. (A) The full-size western blot scans of Dop1 in the locust brains injected with dsGFP and dsDop1. The bands in the blue frames are cropped and presented in Supplementary Figure 3B. (A) The full-size western blot scans of Dop1 in the brains of solitarious and gregarious locusts. The bands in the blue frames are cropped and presented in Figure 1C. Abbreviations: S, solitarious; G, gregarious. M, molecular weight ladder (Thermo Fisher, 26619).

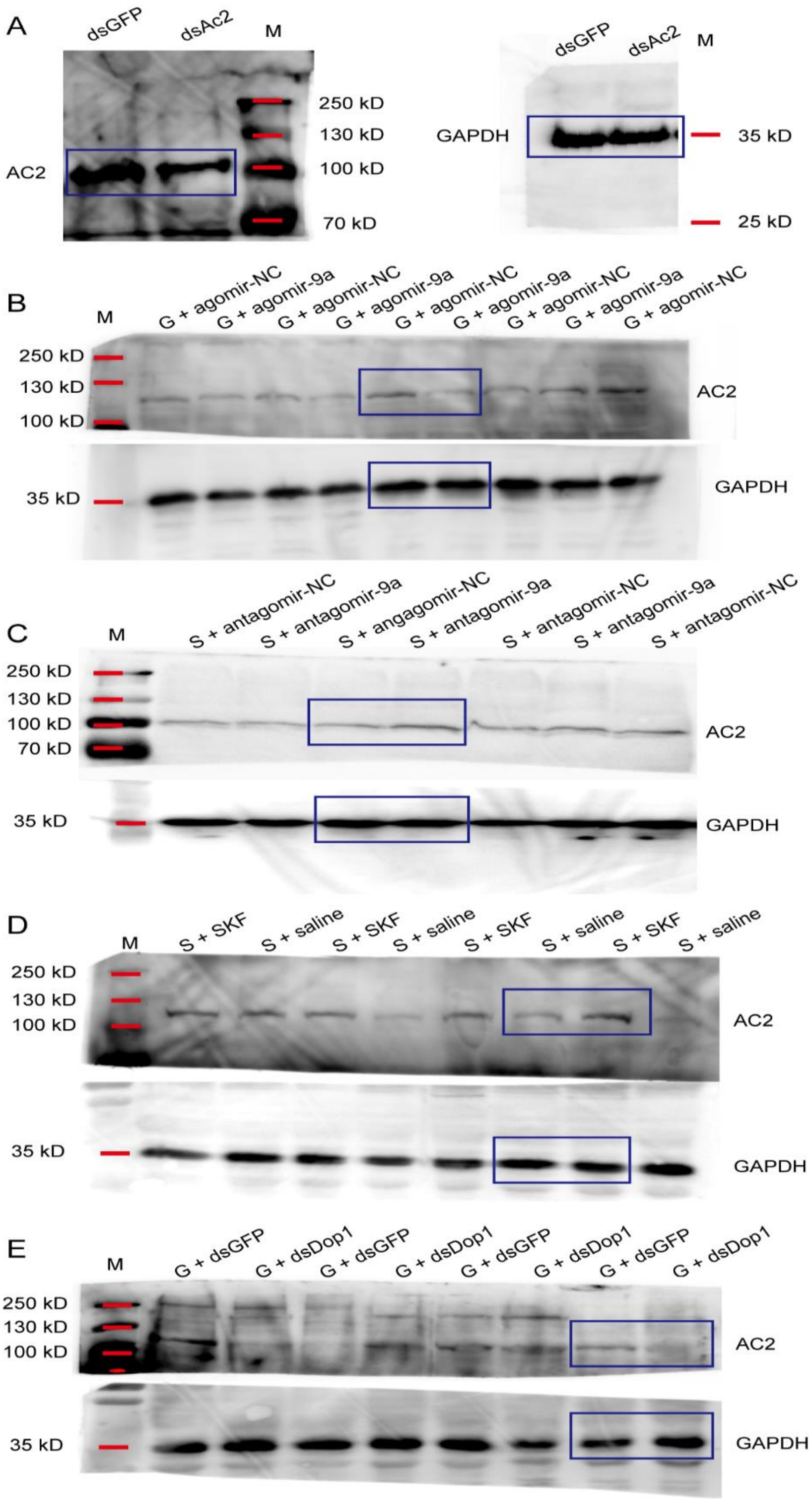

Supplementary Figure 15. The full-size western blot scans of AC2. (A) The full-size western blot scans of AC2 in the locust brains injected with dsGFP and dsAc2. The bands in the blue frames are cropped and presented in Supplementary Figure 10. (B and C) The full-size western blot scans of AC2 in the gregarious (B) and solitarious (C) brains injected with 9a-agomir and 9a-antagomir, respectively. The bands in the blue frames are cropped and presented in Figures 5D and 5F. (D and E) The full-size western blot scans of AC2 in the solitarious (D) and gregarious (E)

brains injected with SKF and dsDop1, respectively. The bands in the blue frames are cropped and presented in Figures 6C and 6D. Abbreviations: S, solitarious; G, gregarious. M, molecular weight ladder (Thermo Fisher, 26619). SKF, SKF38393.

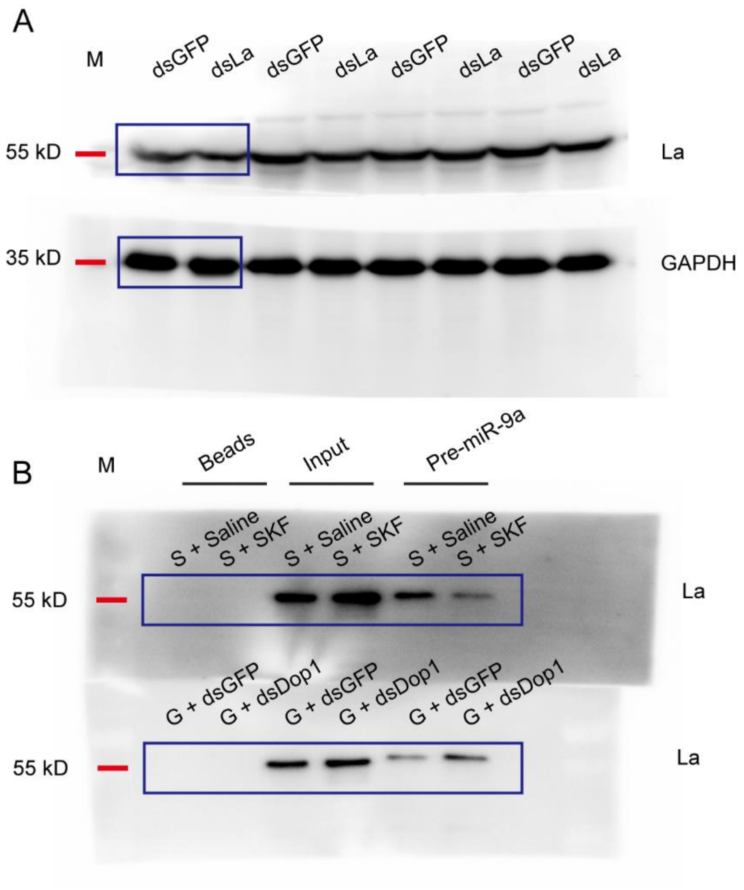

Supplementary Figure 16. The full-size western blot scans of La protein. (A) The full-size western blot scans of La in the locust brains injected with dsGFP and dsLa. The bands in the blue frames are cropped and presented in Supplementary Figure 12B. (B) The full-size western blot scans of La in the solitarious and gregarious brains injected with SKF and Dop1, respectively. The bands in the blue frames are cropped and presented in Figure 8B. Abbreviations: S, solitarious; G, gregarious. M, molecular weight ladder (Thermo Fisher, 26619). SKF, SKF38393.

Supplementary Table 1. Sequences of chemically synthetic RNA

Name	Sequence
Labeled Pre-miR-9a	5'-UUU UUU CUU UGG UUA UCU AGC UGU AUG AGU GCA UGU GAC UGU CAU AAA GCU AGG UUA CCG AAG UUA AG-biotin-3'

Supplementary Table 2. The pre-miR-9a binding protein candidates identified through RNA pull-down and mass spectrometry

Score*	Annotation	*p* value
165	La protein-like protein	3.16228E-17
115	ATP synthase subunit alpha	3.16228E-12
41	Zinc finger protein 294	7.94328E-05
41	Carbonic anhydrase	7.94328E-05
40	RING finger protein 10-like	0.0001
39	Carboxypeptidase B-like	0.000125893
31	Synaptic vesicle glycoprotein 2B	0.000794328
30	Tubulin alpha-1 chain	0.001

* Score indicates ions score that is $-10\log(P)$, where P is the probability that the observed match is a random event. Individual ions score > 36 indicate identity or extensive homology ($p < 0.05$).

Supplementary Table 3. Primer sequences for RNAi.

Genes	Forward primer (5’-3’)	Reverse primer (5’-3’)
Dop1	TCAACGACCTGCTGGGCTA	AAGGGCACCCAGCAGATGA
phf20l1	TTGACGAGAACCTGCCTTATG	TGGTGATCGAAGACTTCTGACA
ac2	GGATGGCACATCACAGAAAC	TATTTACAGCATCACCCCAG
La	GAATGAGGAACGGCGGAAG	TGATTCCGCTGAAGCCTGTT

Supplementary Table 4. The nucleotide sequences for RNAi

Genes	Nucleotide sequences (5’-3’)
Dop1	TCAACGACCTGCTGGGCTACTGGATGTTCGGCGCCCAGTTCTG CGACACCTGGATCGCCTTCGACGTCATGTGCTCCACCGCCTCC ATCCTCAACCTGTGCGCCATCTCGCTCGACAGGTACATCCACA TCAAGGACCCCCTCAGGTACGGGCGGTGGGTGACGCGTCGGG TGGCGGTGGCGAGCATCGCGGCCATCTGGCTGCTGGCGGCGCT CATCTCGTTCCTGCCCATCTCGCTGGGGCTGCACCGGCCGCCG CAGCCGCTCACGTACCACGACGGCGCCACCGAGCACCCCACGT GCGCGCTCGACCTGACGCCCACGTACGCCGTCGTCAGCTCGTG CATCAGCTTCTACTTCCCCACGCTGGTGATGCTGGGCATCTACT GCCGGCTCTACCTGTACGCGCAGAAGCACGTCAAGAACATCCG CGCGGTGACGCGGCCGCTGCCCGCCGCCGCCGCCAGCACCAA CTTCGGCGCGACGGCCGACGCGGGCGACAAGCACCAGCAGCC CATCGCCGCCATGAGCTCGTCGCCCTACCACGTGTCCGACCAC AAGGCGGCCATCACCGTCGGCGTCATCATGGGCGTCTTCCTCA TCTGCTGGGTGCCCTT
phf20l1	TTGACGAGAACCTGCCTTATGGATGGAAGAAATGTGCAGTTCA AAgAAAAGGAGGGTCTTCTATTGGAAAGTGGGATGTGTTCTAC ATCAGTCCACAGGGCAGAAAATTTCGATCACGCAATGAACTG ATGTCTCACCTTAAAAAAGCTGATCAGGTGGTAAGAAAAAAC AAATGTGATTATCCATTACATCAATGCAAGAGAGCAGGAAAA CCAATAGAAAAAGATCTCAGAGCAGCACAAACTACACCTCAA AGACACATGCATTCTGTTGGTTTTAGTAATGATGCACAAAATC AAGGCTTTGGATCTGAACCCCAGAAACGCATTAAGACTTTACT TCCCAAAATCCGGGCTCAGGAAGAAGATAACGAAACAACACA AATGTGTATGACTAAAGGAAAATGTGAAACCAAAGACTGCCC CTCAGCCACTCTGGGCATGCAATCACAGCTGGAATGTCAGAAG TCTtCCGATCACCAA
ac2	GGATGGCACATCACAGAAACATGTGCACTTGTGTTGCTGATGA GTTTTCTTTTCCTGCGAATGCATTTTCAACTCAAACTTCTGATT GGTTGCCTAATCACTGGTGCATATGGCTGTGTCATCTGGATAC TGCAGCCATCCGTATTCCAAAACAGTGAGACATCAAATCCAAA TTTAAAACCACAGGTGGCTCACATCATGTCCATTGTGTTCTTAA CATTCTCACTTCACCTGATAGGCAGGCAGTCAGAGTATATGAA TCGTTTGGATTATCTTTGGAAACGTCAGCTTTCTGAAGAGCAG GATCTATCAGCYACAACTCGTCTAGTGAACAAGATGCTCCTGG AGAACATCTTGCCATCTCATGTTGCTGAACTCTACCTCAATATA GAAAGAACTTCTGATGAGCTGTATCACGAAGAATATGACTCAG TAGCTGTCTTGTTTGCTACAGTTACTGATTATACTCTTTTTGAC TTTGATTATCCTGAAAACAGCAATGAGCTAGTTAGTCTCCGAG TACTCAATGAAATCATATGTGACTTTGATAAGCTACTGTTTGA

	ACCAAATTTCTTACGTGTGGAGAAAATARAAGTTGTTGGATGG<br>ACTTATATGGCTGCTTGTGGCTTGGATCCAGGACGCAGGGACT<br>CAACARCTTCCATTAGCAGGCRATCATTCCCCCAACATGTAGT<br>GTATGTGCTTGCTCGTTTTGCTACAAGGATGATGGCTGCACTA<br>GAAAAAATAAATGAAGAAGGTCAACAATCATACAGGTTGAGA<br>GTGGGTATTTCACATGGCCCAATTACTGCTGGAGTAATTGGAG<br>CACAAAAGCCTCTATATGACATCTGGGGTGATGCTGTAAATA
La	GAATGAGGAACGGCGGAAGGAGCTAATGACTCGCACTCTTTA<br>CTTAAAAGGTTTTCCTGTACAGGACACTACTCTGGACAAACTG<br>TTAGATTTTTTTGCACAGCATTCAACAGTTGAAAATGTTCAGAT<br>GCGTTCGTACAAGGACAAAGCAACAAATAAATTTGTGTTCAAG<br>GGATCTGTGTTTGCCACTTTTCCAACCAAAGAAAAGGCTGAAG<br>AATTTCTGGGCAAGGAAATAAAATATGAAGGAAATGATCTTAT<br>TAAGAAATGGCAATCTGTGTATGTAGAAGAAAAGAAAAAAGA<br>ACGGGAAGAAGCCAAGGCAAAGAGGAGAGGTGGTGCACAAA<br>ATGAGGCTGATAAAAAGGCTCTTCCCAAGGGTGCAATTTTGCA<br>TCTGAAAGGCCTTACTAGTGATATTTCAAGGGAACAAATCAGT<br>GAGAAACTTTCAGAATATGAGGCAGACATAGCTTACATTTCTT<br>ATGACAAAGGGGATGAAGAAGGTTGGATCAGACTCCAAGGCG<br>AAAATTCTACAAAAGCTGTCCTTGAAAAAATTGGGGAGACGA<br>CTTTAGAAATTGGTAATGTAAAAATTGAAGTGAGAGCACTGGA<br>AGGTGAAGAGGAGGAACAATTTCTCAAGAAAACTATGGAGGA<br>CATTCAAAAAAGGAGATTCCACTACAATCAAAACAAACGTGG<br>TCGTAGAGGTGGTGGCAGAGGTGGATTCAGAGGTGACAGGAA<br>AAGAAAACAGGCTTCAGCGGAATCA

Supplementary Table 5. Primer sequences for quantitative PCR.

Genes	Forward primer (5'-3')	Reverse primer (5'-3')
Dop1	GCGCATCGGCAACCTCTTC	GATCCAGGTGTCGCAGAAC
ac2	GGATGGCACATCACAGAAAC	TGCACCAGTGATTAGGCAAC
La	GAATGAGGAACGGCGGAAG	TGTCCTTATACGAACGCATCTG
pfas	CACCGTTGCTTGCTTCTACTA	ACTGCTGGTTACTCTGTTGGA
gart	GTGATTCTTCCTCTGCTTGACT	TGACTTGCCATTACAACTCCAA
purh	GCCTATCAGAGTGGAGTTGAG	ATGGTGGAACAGACGCAAGT
amd1	TTCTAAGGTCTGGTCTGGTTCC	GCAGTTGAGTCCACATAAGGC
sirt3	ATTCCAAGGTCAACTACGCATT	GGCACGGTACTGTCTCCTAA
fgfr3	CACCTCCATCTGCGTCTACC	TGAAGTTGCCGTCACCATCT
unc-89	AGGTGAGACGCTATTCATTCCA	CAGTGCCTCCAACAGTTCTTC
tgmh	GCGTCCAGCGTCTACTACAC	TACTCGTCCACCGTCACCTT
phf20l1	ACAAGGAGCCATTGAGAGTGA	AGGTGAGACATCAGTTCATTGC
RP-49	CGCTACAAGAAGCTTAAGAGG TCAT	CCTACGGCGCACTCTGTTG
miR-9a	ACACTCCAGCTGGGTTCTTTGG TTATCTAGCTGTATG	CTCAACTGGTGTCGTGGAGTCG GCAA
Pre-miR-9 a	AGTGCATGTGACTGTCATAAAG	CTCAACTGGTGTCGTGGAGTCG GCAA
Pri-miR-9 a	GAGCGTCTTCATCCCATCCA	CAACTCCGACACTCCATTAGC
U6	ACACTCCAGCTGGGTCAAAAT CGTGAAGCG	CTCAACTGGTGTCGTGGAGTCG GCAA
18S	ATGCAAACAGAGTCCCGACCA GA	GCGCAGAACCTACCATCGACAG
miR-981	ACACTCCAGCTGGGTTTCGTTG TCGACGAAACCTGCA	CTCAACTGGTGTCGTGGAGTCG GCAA
miR-276	ACACTCCAGCTGGGTTAGGAA CTTCATACCGTGCTCT	CTCAACTGGTGTCGTGGAGTCG GCAA
miR-210-5 p	ACACTCCAGCTGGGTCTTGTGC GTGTGACAGCGGCTAT	CTCAACTGGTGTCGTGGAGTCG GCAA
miR-10-5p	ACACTCCAGCTGGGTTACCCTG	CTCAACTGGTGTCGTGGAGTCG

	TAGATCCGAATTTGT	GCAA
miR-133	ACACTCCAGCTGGGTTTGGTCC CCTTCAACCAGCTGT	CTCAACTGGTGTCGTGGAGTCG GCAA
miR-263a-5p	ACACTCCAGCTGGGTAATGGC ACTGGAAGAATTCACGG	CTCAACTGGTGTCGTGGAGTCG GCAA
miR-193	ACACTCCAGCTGGGTTACTGGC CTGCTAAGTCCCAAG	CTCAACTGGTGTCGTGGAGTCG GCAA
miR-190	ACACTCCAGCTGGGTAGATAT GTTTGATATTCTTGGTTG	CTCAACTGGTGTCGTGGAGTCG GCAA
miR-278-3p	ACACTCCAGCTGGGTTCGGTGG GACTTTCGTCCGTTT	CTCAACTGGTGTCGTGGAGTCG GCAA
miR-2765	ACACTCCAGCTGGGTTGGTAAC TCCACCACCGTTGGC	CTCAACTGGTGTCGTGGAGTCG GCAA
miR-315-5p	ACACTCCAGCTGGGTTTTTGAT TGTTGCTCAGAAAGC	CTCAACTGGTGTCGTGGAGTCG GCAA
miR-8-3p	ACACTCCAGCTGGGTTAATACT GTCAGGTAACGATGT	CTCAACTGGTGTCGTGGAGTCG GCAA
miR-1	ACACTCCAGCTGGGTTGGAAT GTAAAGAAGTATGGAG	CTCAACTGGTGTCGTGGAGTCG GCAA
bantam-3p	ACACTCCAGCTGGGTTGAGATC ATTGTGAAAGCTGATT	CTCAACTGGTGTCGTGGAGTCG GCAA
miR-137	ACACTCCAGCTGGGTTTATTGC TTGAGAATACACGT	CTCAACTGGTGTCGTGGAGTCG GCAA

Supplementary Table 6. Sequences of miRNAs for quantitative PCR.

miRNAs	Sequences (5'-3')
miR-9a	ucuuugguuaucuagcuguaug
miR-981	uucguugucgacgaaaccugca
miR-276	uaggaacuucauaccgugcucu
miR-210-5p	cuugugcgugugacagcggcuau
miR-10-5p	uacccuguagauccgaauuugu
miR-133	uugguccccuucaaccagcugu
miR-263a-5p	aauggcacuggaagaauucacgg
miR-193	uacuggccugcuaagucccaag
miR-190	agauauguuugauauucuugguug
miR-278-3p	ucggugggacuuucguccguuu
miR-2765	ugguaacuccaccaccguuggc
miR-315-5p	uuuugauuguugcucagaaagc
miR-8-3p	uaauacugucagguaacgaugu
miR-1	uggaauguaaagaaguauggag
bantam-3p	ugagaucauugugaaagcugauu
miR-137	uuauugcuugagaauacacgu

Supplementary Table 7. Primer sequences for miRNA overexpression.

miRNAs	Forward primer (5'-3')	Reverse primer (5'-3')
miR-9a	GGTACCCTCCAGCCTCTGTTAGATTGC	CTCGAGGTATTACCAGAGGGTGCAATGT
miR-315-5p	GGTACCGTCTCCTATTTCTTTACCCTTC	CTCGAGGAATAAGGCGATGACGAGTAGG
