# Supplementary material for: Dop1 enhances conspecific olfactory attraction by inhibiting miR-9a maturation in locusts
Source: Nat Commun. 2018 Mar 22;9:1193. doi: 10.1038/s41467-018-03437-z (PMC5864846; doi:10.1038/s41467-018-03437-z)
Supplement: Supplementary file 3 — Description of Additional Supplementary Files(PDF 57 kb) [file 41467_2018_3437_MOESM3_ESM.pdf]

Description of Additional Supplementary Files

File Name: **Supplementary Data 1**
Description: **The potential target genes of miR-9a in the gene database from locust transcriptome.** We employed the algorithms miRanda and RNAhybrid to predict the potential target genes of miR-9a in the gene database from locust brain transcriptome (*Locusta migratoria*). The predicted results are listed in Supplementary Data 1, which includes the accession number of genes in the transcriptome, gene lengths, position of alignment, predict score, gene names, and GO categories.

File Name: **Supplementary Data 2**
Description: **The differentially expressed miR-9a target genes between gregarious and solitarious locusts.** Among the predicted genes of miR-9a (Supplementary Data 1), 157 genes showed significant differences between the gregarious and solitarious phases according to the transcriptome data (Fold change > 1.5). These genes are listed in Supplementary Data 2, which includes the accession number of genes in the transcriptome, gene length, Log2(G/S), *P* value, position of alignment, and annotation.
